# Atopic Eczema: Genetic Analysis of* COL6A5*,* COL8A1*, and* COL10A1* in Mediterranean Populations

**DOI:** 10.1155/2019/3457898

**Published:** 2019-06-04

**Authors:** Claudia Strafella, Valerio Caputo, Giulietta Minozzi, Filippo Milano, Mauro Arcangeli, Nagat Sobhy, Rania Abdelmaksood, Doaa Hashad, Efstratios Vakirlis, Giuseppe Novelli, Raffaella Cascella, Emiliano Giardina

**Affiliations:** ^1^Department of Biomedicine and Prevention, Tor Vergata University, via Montpellier 1, 00133, Rome, Italy; ^2^Laboratory of Genomics Medicine UILDM, Santa Lucia Foundation, via Ardeatina 354, 00142, Rome, Italy; ^3^Department of Veterinary Medicine (DIMEVET), University of Milan, via Celoria 10, 20100, Milan, Italy; ^4^Section of Legal Medicine, Tor Vergata University, via Montpellier 1, 00133, Rome, Italy; ^5^Department of Dermatology, Venereology and Andrology, Faculty of Medicine, University of Alexandria, Khartoum Square, Azzaritta, Alexandria, Egypt; ^6^Clinical Pathology, Faculty of Medicine, University of Alexandria, Khartoum Square, Azzaritta, Alexandria, Egypt; ^7^Department of Dermatology and Venereology, Aristotle University Medical School, 124 Delfon Str, 54643, Thessaloniki, Greece; ^8^Department of Biomedical Sciences, Catholic University Our Lady of Good Counsel, Laprakë, Rruga Dritan Hoxha, 1000, Tirana, Albania

## Abstract

To date, the genes associated with susceptibility to Atopic Eczema (AE) are mainly implicated in immunity, inflammation, and maintenance of skin barrier. Little is known about the possible relationship between genes modulating Extra-Cellular Matrix (ECM) and AE etiopathogenesis. In this regard, the primary objective of the present study has been the investigation of susceptibility biomarkers localized within genes encoding collagen proteins. Several studies have shown that polymorphisms within the genes encoding such proteins may generate abnormal connective tissues, making them more susceptible to mechanical stress, loss of epidermal integrity, and aging. We therefore decided to investigate three polymorphisms located in* COL6A5, COL8A1*, and* COL10A1* as potential susceptibility biomarkers for AE in a cohort of 1470 subjects of Mediterranean origin. The genes of interest have been selected considering that the ECM and immune/inflammatory response are strongly dysregulated in AE and other complex disorders. The study confirmed that the susceptibility to AE depends on a complex interaction between latitude, geographical localization, and the differential distribution of genetic variants among populations exposed to similar environmental factors.

## 1. Introduction

Atopic Eczema (AE, OMIM** #**603165) is one of the most common chronic dermatological diseases caused by the disruption of the skin barrier and the dysregulation of immune and inflammatory responses [1]. Clinical features of the disease include variable age of onset, red blotches, and itchy rashes that quickly develop into painful papule, xerosis, erythematous lesions with increased transepidermal water loss, and disruption of the epidermal barrier function [2]. AE affects approximately 1-3% of the adult population and 15-20% of children, depending on population and geographical localization. In particular, higher prevalence (≥15%) of disease has been observed among populations living in Northern Europe (England, Denmark, Norway, Ireland) and Asia (Japan, Korea, Taiwan) while Southern Europe and Mediterranean areas (Turkey, Malta, Croatia, Spain, Italy, Greece, Egypt) reported lower prevalence (≤5%) rates [3]. The variable prevalence across the world might reflect the influence of different UV exposure with respect to latitude and geographic localization. This variability may in turn be responsible for a different genetic adaptation to local environment which ultimately determines a population-specific susceptibility to AE. Genetic adaptation is meant as the positive/negative presence for variants localized within genes involved in the regulation of skin permeability and response to UV radiations. An excellent example of this condition is represented by the mutational spectrum of the* FLG* (1q21) gene associated with AE. Interestingly, the frequency of* FLG* mutations showed a heterogeneous distribution between Northern European and Mediterranean subjects (0% in Mediterranean areas vs. 10% in Northern Europe) [4, 5].

However, although* FLG* mutations are one of the most predisposing factors to the disease, other genetic variations can also be critical for the onset and severity of AE. The presence of several susceptibility loci can be easily explicated by the multifactorial etiology of the disease that depends on the complex interaction between environmental factors (toxin, skin irritants, climate pollutants, and microorganisms) and multiple genes variations. To date, more than 30 loci have been associated with a higher risk of AE [6–8]. All of the genes which have been identified so far are essentially implicated in the immune and inflammatory responses and in the maintenance of skin barrier. Little is known about the possible relationship between genes modulating Extra-Cellular Matrix (ECM) and AE etiopathogenesis. In this regard, the primary objective of the present study has been the investigation of susceptibility biomarkers localized within genes encoding collagen proteins. Collagen is a fibrous protein primarily involved in the formation and maintenance of structural components such as cartilage, bone, epidermis, cornea, and blood vessels. To date, at least 28 subtypes of collagen are known to be differentially expressed in body tissues. Several studies have shown that polymorphisms within the genes encoding such proteins may generate abnormal connective tissues which may be more susceptible to mechanical stress, loss of epidermal integrity, and aging [9–12]. We therefore decided to investigate three polymorphisms located in* COL6A5* (3q22.1),* COL8A1* (3q12.1), and* COL10A1* (6q22.1) as potential susceptibility biomarkers for AE in a large cohort of Mediterranean origin. The genes of interest have been selected considering that they are involved in the organization of ECM and immune/inflammatory responses, both of which are known to be strongly dysregulated in AE and other complex disorders.

## 2. Material and Methods

### 2.1. Study Cohorts

The study was performed on 428 patients affected by Atopic Eczema (AE) from Italy, Egypt, and Greece (collectively referred to as Mediterranean populations). In particular, 285 Italian individuals were recruited from the Immunoallergology Unit of San Pietro Fatebenefratelli Hospital of Rome and IDI (Istituto Dermopatico dell'Immacolata, Rome); 87 Egyptian individuals were enrolled from the University of Alexandria and 56 Greek individuals from the Aristotle University Medical School, Thessaloniki. The clinical diagnosis fulfilled the diagnostic criteria of the “UK Working Party for Atopic Dermatitis” [13] and was assessed either by expert dermatologists or by pediatric allergologists. Concerning control subjects, 992 healthy individuals of Mediterranean ancestry were enrolled from “Tor Vergata” General Hospital of Rome. In addition, 50 Greek healthy subjects from Aristotle University Medical School (Thessaloniki, Greece) were subsequently included in this study. The diagnosis of AE was excluded for all control subjects at the time of recruitment. The study was approved by the Ethics Committee of each participating institute and was performed according to the Declaration of Helsinki. All participants provided signed informed consent.

### 2.2. DNA Extraction and Genotyping Analysis

Whole blood samples were taken from patients and controls to extract genomic DNA. DNA extraction was performed by the EZ1 Advanced XL automated extractor and the EZ1 DNA Blood 200 *μ*l Kit (Qiagen). The genotyping analysis was performed on a 7500 Fast Real Time PCR (Applied Biosystems) according to manufacturer's instructions. Specific predesigned TaqMan assays were utilized for* COL6A5* (rs12488457, A/C),* COL8A1* (rs13081855, G/T), and* COL10A1 *(rs3812111, A/T) genotyping. The results were interpreted using Sequence Detection System 2.1 software (Applied Biosystems). Each Real Time PCR run was performed using a negative control and three positive control samples previously confirmed by direct sequencing (BigDye Terminator v3.1, BigDyeXTerminator) on ABI3130xl (Applied Biosystems). Direct sequencing of the samples was conducted following the manufacturer's indications.

### 2.3. Biostatistical Analysis

The genotyping results were subjected to biostatistical analysis to evaluate the association with AE. Allele and genotype frequencies of the analyzed SNPs were calculated by direct counting. Hardy-Weinberg Equilibrium (HWE) was tested at each locus, by comparing the observed genotype frequencies with those expected under HWE. Data were considered in HWE for* p*>0.05. The association of the SNPs was measured by calculating the p-value (*p*) through a contingency table and the *χ*^2^ test. The cut-off for statistical significance was set at a* p*<0.05. The strength of association was determined by calculating the Odd Ratio (OR) and a 95% confidence interval. All the statistical analyses were performed using the SPSS program, ver. 23 (IBM Corp).

### 2.4. Bioinformatic Analysis

Bioinformatic tools were employed to assess the “*in silico”* functional role of the analysed SNPs. In particular, Mutation Taster, Polyphen-2 and ExPaSy-Prosite [14–16] were used to predict the impact of the associated SNPs on protein structure and function. Human Splicing Finder was interrogated to assess whether the analysed SNPs could alter the splicing activity. All of these tools normally utilize a set of algorithms and database to calculate a reliable score and predict the effect size of the variants taken into consideration. Gene enrichment tools available from DAVID (Database for Annotation, Visualization and Integrated Discovery) [17, 18] and KEGG (Kyoto Encyclopaedia of Genes and Genomes) [19] were used to investigate gene-gene interactions and their potential involvement in disease-associated molecular pathways. These enrichment tools incorporate data from different public resources and provide information about the possible involvement of the genes of interest in biological and molecular pathways.

The* COL8A1* (3q12.1) and* COL6A5* (3q22.1) genes are both located on the long arm of chromosome 3; therefore, we decided to evaluate the Linkage Disequilibrium (LD) pattern in our Mediterranean samples. The LD analysis was performed on the PhAT (Pharmacogenetics of Asthma Treatment) Website using the LD Plotter online tool (http://pharmgat.org/Tools/pbtoldplotform). The LD Plotter tool allows conversion of a* prettybase* format file into a plot showing pairwise LD. The LD pattern has been measured in D' which exploits allele frequencies to provide a scale of values ranging from 0 (indicating high recombination between the markers) to 1 (meant as low or absent recombination between the markers). The results have been displayed in numerical LD values and color scheme.

## 3. Results

A total of 428 Mediterranean patients (285 Italians, 87 Egyptians, and 56 Greeks) with a diagnosis of AE were recruited for the study. In addition, 992 healthy individuals of Mediterranean ancestry and 50 Greek subjects were utilized as control cohort. Genomic DNA was obtained from all the patients' samples. Successively, the samples were genotyped for the SNPs of interest (rs12488457, rs13081855, and rs3812111) by Real Time PCR and TaqMan chemistry. The genotyping results were subjected to biostatistical and bioinformatic analysis to evaluate the association with AE.

### 3.1. Biostatistical Results

The genotyping results related to rs13081855 (G/T,* COL8A1*) were subjected to biostatistical analysis, revealing a positive association with AE in Mediterranean cohorts [*p=*2.09*∗*10^−5^, OR(T) = 1.88 (CI95%: 1.40 - 2.53)], even taking into account population-specific subgroups [Egyptian cohort:* p=*0.03, OR(T) = 1.77 (CI95%: 1.04 - 2.99) and Italians:* p=*1.32*∗*10^−6^, OR(T) = 2.20 (CI95%: 1.58 - 3.05)]. In this context, the Greek group represents the only exception to this result, since the rs13081855 was not associated with AE (Table 1). The allelic distribution of rs13081855 within Greek patients is highly similar to the frequency observed among control subjects. This result has been further confirmed by comparing the frequencies found in healthy individuals of Greek origin (Table 2). Given these data,* COL8A1 *may be considered as a population-specific biomarker for AE susceptibility although this association should be replicated in other cohorts as well.

The rs12488457 (A/C,* COL6A5*) did not reveal any significant association among Mediterranean populations, except for the Greek cohort. In fact, the biostatistical analysis performed on the Greek group compared to the overall control subjects reported a* p=*0.019 for the rs12488457 (A/C) and an OR(C)=1.62 (CI95%:1.08-2.44) (Table 1). These results may be explained considering the allele frequencies among Mediterranean patients, overall control subjects, and the Greek subset of patients. A similar allele distribution was observed among Mediterranean and control cohorts, in contrast to the different frequencies reported in the Greek population (Table 1). However, a subsequent analysis considering only patients and controls of Greek origin resulted to be not significant (Table 2), suggesting that* COL6A5* is not a susceptibility factor for AE in Mediterranean populations, although the Greek cohort differs from the other Mediterranean groups as a result of a variable allele distribution of the SNP of interest.

The biostatistical evaluation of the results obtained from rs3812111 (A/T,* COL10A1*) genotyping did not report any significant association with AE among Mediterranean populations. As for* COL6A5*, only the Greek group revealed significant values [*p*=0.018, OR(T) = 1.69 (CI95%: 1.09 - 2.64)], although this result was not confirmed in the subsequent analysis considering only Greek control individuals (Tables 1 and 2). Even in this case, the frequency distribution of rs3812111 in Greek population differed from the frequencies observed in the other analysed sample cohorts (Table 1). Therefore, these results suggest that* COL10A1 *does not represent a susceptibility factor for AE in Mediterranean populations, although the Greek cohort displayed his own peculiar allele frequency distribution compared to other groups of Mediterranean ancestry.

### 3.2. Bioinformatic Results

Bioinformatic analysis was performed to assess the impact of the SNPs on protein structure and function as well as to investigate the functional role of the analysed genes in biological processes. In particular, the rs13081855 is an intronic variant within* COL8A1 *coding for the *α*1 chain of Collagen VIII. Interrogation of Mutation Taster and Human Splicing Finder did not provide evidence of splicing alteration for this SNP. GO analysis, performed on DAVID, reported that the gene is involved in angiogenesis pathways.

The rs12488457 is a missense Thr/Pro polymorphism located into the 9th exon of* COL6A5 *that is a gene encoding the *α*5 chain of Collagen VI. Mutation Taster and ExPaSy-ProSite outcomes reported that the SNP could potentially affect protein structure, by altering specific domains or protein features, namely, VWFA7 and nonhelical region, respectively. In addition, a significant result was also predicted by Polyphen-2, which reported a score of 0.987 consistent with a strong impact of the variant, considering a significance range between 0 (no impact) and 1 (maximum impact). Moreover, Mutation Taster and Human Splicing Finder indicated that the variant might also alter a regulatory site and, consequently, influence the splicing mechanisms of COL6A5 protein.

Gene Ontology (GO) enrichment analysis performed on DAVID showed that* COL6A5* is involved in cell adhesion mechanisms, collagen synthesis, and catabolism. In addition, KEGG reported the COL6A5 involvement in focal adhesion and Pi3k/Akt pathways.

Since* COL8A1* (3q12.1) and* COL6A5* (3q22.1) genes are both located on the long arm of chromosome 3, the LD analysis was performed in order to assess the existence of recombination between the SNPs of interest. As expected, the LD pattern was excluded because obtained D' values range from 0 to 0.22 in our Mediterranean cohort.

Finally, the rs3812111 is an intronic variant located into* COL10A1*, which is essential for the synthesis of *α*1 chain of Collagen X. The interrogation of Mutation Taster and Human Splicing Finder did not report significant splicing changes for this SNP compared to the wild-type sequence. On the basis of the results collected on DAVID,* COL10A1* mainly exerts its role in bone and cartilage metabolism processes as well as in the organization and remodelling of ECM.

## 4. Discussion

The main purpose of the study was the investigation of the relationship between dysregulation of ECM and the susceptibility to AE. In particular, genes encoding collagen proteins have been analyzed to identify candidate biomarkers able to discriminate individuals with a higher risk of AE with respect to the general population. Considering the high heterogeneity of the disease due to different susceptibility factors in terms of geographic localization, latitude, and frequency distribution of disease-associated genetic variants, a cohort of 1470 subjects of Mediterranean origin has been genotyped for three polymorphisms located on the* COL6A5*,* COL8A1*, and* COL10A1* genes.

The statistical analysis identified a positive association of* COL8A1* (rs13081855, G/T) with AE in Mediterranean populations, except for the Greek group, as the allelic distribution of the SNP within Greek patients is similar to the frequency observed among control subjects. The subsequent analysis on the three different Mediterranean cohorts confirmed this result, suggesting that* COL8A1* may be considered as a population-specific biomarker for AE (see Table 1). It would be interesting to replicate these data even in other cohorts of Mediterranean ancestry. On this subject, bioinformatic investigation performed on DAVID highlighted the involvement of the gene in disease-associated cellular and molecular pathways. In fact, the GO enrichment tool of DAVID reported that COL8A1 protein contributes to maintain the integrity and structure of vessel walls, the ECM remodelling activity and to induce angiogenesis. On this subject,* COL8A1* has been extensively investigated in other multifactorial disease, especially in Age-Related Macular Degeneration (AMD). The gene has been associated with the neovascular forms of AMD that is characterized by the abnormal growth of blood vessels beneath the retina. In this context,* COL8A1* has been found to be associated with a higher risk of neovascular AMD in Italian population [20, 21]. Moreover, bioinformatic analysis, performed by DAVID and KEGG databases, revealed a significant interaction with* VEGFA* that is one of the main proangiogenic factors and it may be thereby investigated as an additional player in the etiopathogenesis of AE. Supporting this hypothesis, the alteration of keratinocytes metabolism is known to be responsible for an overproduction of proangiogenic factors which stimulate the growth of blood vessels towards the epidermis that is normally nonvascularized. The originated new vessels are characterized by tortuous dermal capillary loops which may contribute to the disease exacerbation in a similar way to that described for lesions occurring in patients with Psoriasis (Ps) and Psoriatic Arthritis (PsA) [22]. Given this result, it would be interesting to validate* COL8A1* as a susceptibility factor for Ps and PsA as well as studying this gene as a candidate therapeutic target.

The genotyping analysis of* COL6A5* (rs12488457, A/C) reported similar allelic frequency distributions in our Mediterranean cohort and control subjects, resulting thereby in a nonsignificantly associated marker with AE. However, the subsequent analysis performed on single populations of Mediterranean ancestry (Italian, Egyptian and Greek) revealed a significant association only for Greek patients compared to general control subjects. Successively, biostatistical analysis between patients and controls of Greek origin gave nonsignificant results, suggesting that* COL6A5* does not represent a susceptibility factor for AE in Greek population as well as in the other Mediterranean groups. Supporting this negative result, the cellular and molecular functions of COL6A5 protein have been evaluated by means of bioinformatics approaches. COL6A5 is expressed in the dermoepidermal junctions, especially within the dermal papillae where it takes part in the metabolic activities of collagen, cellular adhesion, and interaction of ECM proteins localized at dermal level. The cellular localization and function of COL6A5 may explain the lack of association with AE. In fact, the peculiar lesions observed in AE patients normally affect the outer layers of skin without undermining dermoepidermal junctions. However, the association of* COL6A5* with AE is controversial in literature as some works described it as a susceptibility factor among non-Mediterranean populations [23] while a case-control study including patients from Germany and other European Countries [24, 25] did not confirm the previous results. The real impact of* COL6A5* gene on disease etiopathogenesis needs therefore to be clarified by further investigations. As for* COL6A5*, statistical association analysis did not report any significant association between* COL10A1* (rs3812111, A/T) and AE among Mediterranean populations, except for the Greek group, which showed to be significantly associated with respect to general control subjects. This data was not confirmed by comparing Greek patients and Greek control individuals, suggesting that* COL10A1* is not a susceptibility factor for AE in Mediterranean populations. Concerning the molecular function of COL10A1 protein, bioinformatic analysis revealed its implication in ECM organization, maintenance of joints, and bone homeostasis. Therefore, the role of COL10A1 explains the lack of association with AE, considering that the disease is normally characterized by the alteration of inflammatory/immune response and epidermal differentiation mechanisms without undermining bone tissue.

Altogether, our data indicate* COL8A1* as a novel susceptibility factor for AE in Mediterranean populations, except for the Greek cohort (Table 1). Interestingly, this group of patients showed a different frequency distribution compared to the other analyzed cohorts of Mediterranean ancestry. This divergence may be explained by the presence of a specific allelic architecture within the Greek area probably due to a positive/negative selection of genetic variants in response to the adaptation to the local environment. Otherwise, the different results may be due to the small sample-size available for the study, which may have misled the real frequency distributions within Greek population. The results obtained in the Greek cohort have to be therefore validated on a larger sample-size, in order to evaluate the actual relationship between genetic variants in collagen genes and the susceptibility to AE in this population. Similarly, our results should also be replicated in other worldwide populations characterized by different exposures to environmental factors which may thereby interact with specific genetic features and confer a higher/lower risk of AE.

Over the replication of our data on a larger Greek cohort and other populations, future perspectives include the evaluation of the association of* COL6A5*,* COL8A1,* and* COL10A1* in other skin-related disorders, such as Ps and PsA that share similar etiopathogenetic pathways observed in AE. In fact, the possibility of identifying differential biomarkers for the susceptibility to AE, Ps, or PsA could be useful for revealing novel phenotype-genotype correlations and highlighting common and differential etiopathogenetic pathways underlying these disorders.

## 5. Conclusions

The study was mainly addressed to investigate the relationship between dysregulation of ECM and the susceptibility to AE. The genes of interest have been selected considering their involvement in the organization of ECM and immune/inflammatory responses, both of which are known to be strongly dysregulated in AE and other complex disorders. Particular attention was given to collagens genes, since variations within their sequence may contribute to generating abnormal connective tissues which are more susceptible to mechanical stress, loss of epidermal integrity, and aging. In the present study, three polymorphisms located in* COL6A*,* COL8A1*, and* COL10A1* were investigated as potential susceptibility biomarkers for AE in a large cohort of Mediterranean origin. The obtained results confirmed that the susceptibility to AE depends on a complex interaction between latitude/geographical localization and the differential distribution of genetic variants among populations exposed to similar environmental factors. This relationship can be clinically useful to stratify patients on the basis of their individual genetic features and the exposure to environmental factors that can impact not only the onset/progression of disease but also the response to topic treatments and, ultimately, the quality of life of patients suffering from AE.

## Figures and Tables

**Table 1:** Biostatistical results obtained by analysis of Mediterranean and control subjects.

Gene (SNP)	Population (N)	Genotypes (Frequency)	Alleles (Frequency)	*P*	OR (95%CI)
*COL6A5 *(rs12488457, A/C)	Mediterranean (428)	AA: 85 (0.20) AC: 207 (0.48) CC: 136 (0.32)	A: 377 (0.44) C: 479 (0.56)	ns	-
	Egyptian (87)	AA: 11 (0.13) AC: 46 (0.53) CC: 30 (0.34)	A: 68 (0.39) C: 106 (0.61)	ns	-
	Italian (285)	AA: 68 (0.24) AC: 137 (0.48) CC: 80 (0.28)	A: 273 (0.48) C: 297 (0.52)	ns	-
	Greek (56)	AA: 6 (0.11) AC: 24 (0.43) CC: 26 (0.46)	A: 36 (0.32) C: 76 (0.68)	0.019	C: 1.62 (1.08-2.44)
	Control Subjects (832)	AA: 150 (0.18) AC: 424 (0.51) CC: 258 (0.31)	A: 724 (0.44) C: 940 (0.56)		

*COL8A1* (rs13081855, G/T)	Mediterranean (398)	GG: 314 (0.790) GT: 81 (0.203) TT: 3 (0.007)	G: 709 (0.89) T: 87 (0.11)	2.09*∗*10^−5^	T: 1.88 (1.40-2.53)
	Egyptian (87)	GG: 69 (0.79) GT: 18 (0.21) TT: 0 (0.00)	G: 156 (0.90) T: 18 (0.10)	0.03	T: 1.77 (1.04-2.99)
	Italian (255)	GG: 194 (0.86) GT: 58 (0.23) TT: 3 (0.01)	G:446 (0.87) T: 64 (0.13)	1.32*∗*10^−6^	T: 2.20 (1.58-3.05)
	Greek (56)	GG: 51 (0.91) GT: 5 (0.09) TT: 0 (0.00)	G: 107 (0.96) T: 5 (0.04)	ns	-
	Control Subjects (883)	GG: 778 (0.881) GT: 102 (0.116) TT: 3 (0.003)	G: 1658 (0.94) T: 108 (0.06)		

*COL10A1* (rs3812111, A/T)	Mediterranean (402)	AA: 53 (0.13) AT: 191 (0.48) TT: 158 (0.39)	A: 297 (0.37) T: 507 (0.63)	ns	-
	Egyptian (87)	AA: 10 (0.11) AT: 47 (0.54) TT: 30 (0.35)	A: 67 (0.39) T: 107 (0.61)	ns	-
	Italian (259)	AA: 41 (0.16) AT: 121(0.47) TT: 97 (0.37)	A: 203 (0.39) T: 315 (0.61)	ns	-
	Greek (56)	AA: 2 (0.04) AT: 23 (0.41) TT: 31 (0.55)	A: 27 (0.24) T: 85 (0.76)	0.018	T: 1.69 (1.09-2.64)
	Control Subjects (992)	AA: 135 (0.14) AT: 425 (0.48) TT: 432 (0.39)	A: 695 (0.35) T:1289 (0.65)		

**Table 2:** Biostatistical results obtained by analysis on Greek case and control subjects.

Gene (SNP)	Population (N)	Genotypes (Frequency)	Alleles (Frequency)	*P*	OR (95%CI)
*COL6A5 *(rs12488457, A/C)	Greek (56)	AA: 6 (0.11) AC: 24 (0.43) CC: 26 (0.46)	A: 36 (0.32) C: 76 (0.68)	ns	-
	Greek Controls (50)	AA: 8 (0.16) AC: 18 (0.36) CC: 24 (0.48)	A: 34 (0.34) C: 66 (0.66)		

*COL8A1* (rs13081855, G/T)	Greek (56)	GG: 51 (0.91) GT: 5 (0.09) TT: 0 (0.00)	G: 107 (0.96) T: 5 (0.04)	ns	-
	Greek Controls (50)	GG: 40 (0.80) GT: 10 (0.20) TT: 0 (0.00)	G: 90 (0.90) T: 10 (0.10)		

*COL10A1* (rs3812111, A/T)	Greek (56)	AA: 2 (0.04) AT: 23 (0.41) TT: 31 (0.55)	A: 27 (0.24) T: 85 (0.76)	ns	-
	Greek Controls (48)	AA: 6 (0.125) AT: 18 (0.375) TT: 24 (0.500)	A: 30 (0.31) T: 66 (0.69)		

## Data Availability

The data used to support the findings of this study are included within the article.
